# Antiviral drugs against hepatitis C virus

**DOI:** 10.1186/1479-0556-9-11

**Published:** 2011-06-23

**Authors:** Sidra Rehman, Usman A Ashfaq, Tariq Javed

**Affiliations:** 1 Division of Molecular Medicine, National Centre of Excellence in Molecular Biology, University of the Punjab, Lahore, Pakistan

## Abstract

Hepatitis C virus (HCV) infection is a major worldwide problem causes acute and chronic HCV infection. Current treatment of HCV includes pegylated interferon-α (PEG IFN- α) plus ribavirin (RBV) which has significant side effects depending upon the type of genotype. Currently, there is a need to develop antiviral agents, both from synthetic chemistry and Herbal sources. In the last decade, various novel HCV replication, helicase and entry inhibitors have been synthesized and some of which have been entered in different phases of clinical trials. Successful results have been acquired by executing combinational therapy of compounds with standard regime in different HCV replicons. Even though, diverse groups of compounds have been described as antiviral targets against HCV via Specifically Targeted Antiviral Therapy for hepatitis C (STAT-C) approach (in which compounds are designed to directly block HCV or host proteins concerned in HCV replication), still there is a need to improve the properties of existing antiviral compounds. In this review, we sum up potent antiviral compounds against entry, unwinding and replication of HCV and discussed their activity in combination with standard therapy. Conclusively, further innovative research on chemical compounds will lead to consistent standard therapy with fewer side effects.

## Introduction

**Figure 1:**
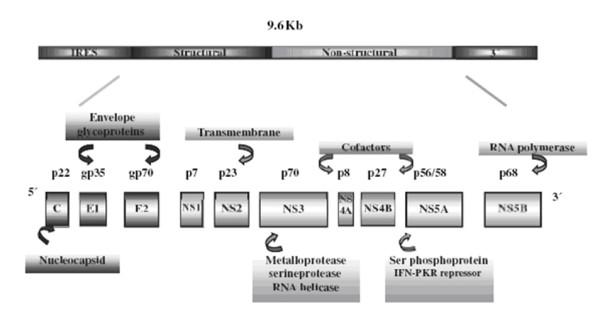
**HCV structure **: HCV enclosing a single stranded RNA of 9.6 kb. The genome carries a single long open reading frame (ORF) which on processing forms a polyprotein that is proteolytically cleaved into distinctive products. The HCV polyprotein is cleaved co- and post-translationally by cellular and viral proteinases into 10 different products, with the structural proteins located in the amino- terminal one-third and the nonstructural (NS) replicative proteins in the rest. (5)

HCV belonging to the family Flaviviridae signifies to be an entire global dilemma which parades the variability of genome translated into six genotypes and more than 80 subtypes. HCV has infected 200 million people worldwide [1], of which 10 million individuals (6% of the population) have been spotted in Pakistan [2]. HCV was firstly recognized in 1989 [3], comprising of 9.6 kb positive sense genome. It encodes a single polyprotein precursor of 3010 amino acids having an internal ribosome entry site at 5' untranslated region (UTR), vital for the translation. This polyprotein precursor is co-translationally processed by cellular and viral proteases into three structural proteins (core, E1 & E2) and seven non-structural proteins (P7, NS2, NS3, NS4A, NS4B, NS5A & NS5B) [4] (Figure 1).

HCV infection is generally going to be clinically imperceptible after 3-12 weeks of incubation [5]. Currently, it is estimated that 50-80% of patients have successively infected with chronic infection and 2-5% have developed hepatocellular carcinoma per annum. HCV has the capacity to stimulate immunopathological effects, engendering reactive oxygen species (ROS) impend indirectly fibrogenetic effects [6] leading to steatosis and cirrhosis [7]. HCV infection commences while interaction of virions instigate with various cellular receptors [8]. After internalization of virions by clathrin-mediated endocytosis [9,10], HCV RNA is being released into cytosol followed by translation and progression to viral proteins. A large number of viral progeny particles are released through the secretory pathway after assemblage of new genomic RNA and structural proteins.

Recently, there is no precise antiviral regime for the deterrence of HCV infection. Nevertheless, current standard treatment pegylated interferon-α (PEG IFN- α) in combination with ribavirin (RBV) have been employed with certain side effects and slow response rate especially in patients infected with HCV genotype 1a and1b [11,12]. Now a day, various novel antiviral inhibitors have been accounted showing a promising approach against HCV.

## Antiviral Drugs & Their Mode of Action

Mainly, an array of attempts has been focused especially on these targets: NS3-4A serine protease, RNA helicase activity of NS3, NS5B RNA-dependent RNA polymerase (RdRp), agents that enhance immunomodulatory activity by developing HCV replicon system. Likewise, the HCV replicon system illustrated an exclusive drug-screening system for antiviral compounds exhibiting the potency to hamper the viral enzymes and HCV RNA replication process in cellular environment. However, antiviral compound-resistant mutations are credibly arising in viral genome due to high heterogeneity while developing the specific HCV protease and polymerase inhibitors [13]. Various efforts are being made in screening antiviral compounds against different HCV replicon systems [14-16].

### Inhibitors of HCV RNA Replication

HCV replication is instigated by the formation of replicase complex which is allied with intracellular membrane containing cellular proteins. Replicase complex consists of cleavage products of HCV polyprotein precursor especially NS3-5B which play an important role in replication. Along with these proteins and cis acting RNA elements, various host factors are also involved in HCV RNA replication [17-19]. NS5B is the RNA-dependent RNA polymerase (RdRp) which can start RNA synthesis de novo. RdRp activity is shown to be enhanced by interacting with cyclophilin B and viral factors such as NS3 and NS5A. A negative-strand copy of viral genome is primarily produced by NS5B RdRp. *In-vitro *this enzyme has a preference for primer-dependent RNA synthesis, either by elongation of a primer hybridized to an RNA homopolymer or through a copy-back mechanism while exploiting heteropolymeric templates [20,21]. NS3 protein possesses helicase, protease and RNA triphosphatase activity. Even though NS3 exhibits innate proteolytic activity, NS4A cofactor is required for the cleavage of polyprotein. Due to vague understanding of helicase enzymology, NS3 helicase is a hard-hitting target for drug designing [22].

The illustration of HCV replication is made possible by the development of HCV cell culture system. First HCV replicon was generated in human hepatome cell line (Huh-7) having genotype 1b subgenomic RNA along with 5' UTR, neomycin phosphotransferase gene (NPT), internal ribosome entry site (IRES) of encephylomyocarditis virus (EMCV)-HCV NS3-4A-4B-5A-5B-HCV 3' UTR. RNA replication, virus-host relations, screening of antiviral drugs and their mechanism are best studied by the replicon system [23]. Nucleosides inhibitors (NI) as well as non nucleoside inhibitors (NNI) of HCV NS5B RdRp have been appraised. Specifically Targeted Antiviral Therapy for hepatitis C (STAT-C) approach is now being currently used to develop drugs that basically target specific enzymes involved in HCV replication. STAT-C drugs such as polymerase and protease inhibitors are presently accessible only in different phases of clinical trials.

Debio 025, a non-immunosuppressive cyclosporine (Cs) analogue, is found to exhibit novel inhibition of HCV replication when used alone or in combination with STAT-C inhibitors. To date, Debio 025 was pooled with RBV, VX-950 a protease inhibitor, 2'-*C*-Methylcytidine (2'-*C*-MeCyt) a NI and JT-16 a NNI. All these amalgamations produced additive antiviral effects showing the lack of interference with biological activity of each other which may either, resulted in synergistic or antagonistic effect. Combinations of low dose of Debio 025 with specific STAT-C inhibitors also prevent the progress of STAT-C inhibitor-resistant variant; hence, it may prove to be a striking antiviral agent for the treatment of HCV infection [24]. In phase II study of clinical trials, it is being found that Debio 025 is a novel HCV inhibitor by binding to cyclophilin A (CyP) in domain II of NS5A which is crucial for replication. Resistance outline of Debio 025 presents a distinctive selection in treating chronic HCV infection, both as the backbone of forthcoming combination therapy with other compounds for treatment and as save therapy for patients anchoraging resistance mutations to other anti-HCV agents [25].

Combined effect of HCV-796 (an NNI of HCV NS5B) and boceprevir SCH 503034 (an inhibitor of NS3 serine protease) was tested to check their competence for producing resistant replicon variants. Conclusively, substantial antiviral efficiency was assessed in combinational treatment along with low emergence rate of viral variants with reduced propensity. This study offers a basis for the clinical estimation of three-part combination of PEG IFN-α, boceprevir and HCV-796 [26].

Since RdRp is deficient in proof reading activity during replication so error rate is very high, resulting in an ample genetic diversity in viral populace within each patient. This diversification in genome is directly related with the low response to HCV RdRp inhibitors especially in patients of genotype 1a and 1b [27,28].

PF-00868554, an NNI of HCV RdRp, has demonstrated both specificity and capability for 1a and 1b genotypes including clinical and laboratory isolates. During *in-vitro *resistance study of PF-00868554, amino acid (AA) changes were recognized at the allosteric site of the polymerase, comprising M423T/V/I, M426T, and I482T, but switching at M423 resulted in relatively much resistance than others. Notably, replicons enclosing these resistance changes have found no cross-resistance with IFN and other polymerase inhibitors, sustaining the make use of PF-00868554 in combination therapies [29].

Antiviral activity of 7-deazaneplanocin A (7-DNPA) is reported against HCV with low cell toxicity in HCV RNA replicon system in Huh-7 cell line. Anti-HCV activity of 7-DNPA is comparable to the 2'-*C*-Me-cytosine (2'-*C*-Me- C) or 2'-F-*C*-Me-cytosine (2'-F-*C*-Me-C) which were used as positive controls, by quantifying through real time RT-PCR. Various derivatives of 7-DNPA are synthesized by replacing different functional groups at 7-position of DNPA, of which some are devoid of anti-HCV activity while others such as 7-carboxamide derivative exhibiting significant antiviral activity against HCV [30].

Combinations of nucleoside analogues β-D-2'-*C*-methylcytidine (2'-*C*-MeC; NM-107) or β-D-2'-deoxy-2'-fluoro-2'- *C*-methylcytidine (2'-F-*C*-MeC; PSI-6130) with interferon-α 2b (IFN-α2b) plus ribavirin (RBV) were assessed in subgenomic HCV relicon. β-D-2'-*C*-methylcytidine (2'-*C*-MeC; NM-107) was the first nucleoside HCV inhibitor. Triple combination of valopicitabine (NM-283), the 3'-valine ester of β-D-2'-*C*-methylcytidine (2'-*C*-MeC; NM-107) along with IFN and RBV resulted in 70% decline in viral load, but NM-283 was interdicted due to gastrointestinal side effects [31]. The distinction of combination index (CI) of two sets of three combinations pointed towards striking synergism of NM-107 with IFN + RBV than PSI-6130 combination to inhibit HCV RNA replication [31].

Nitazoxanide (NTZ) was originally ascertained for intestinal protozoan infection; later on its antiviral characteristics were established. NTZ, and its metabolite, tizoxanide (TIZ), exhibit constancy with resistance in HCV replicon containing cell line bestowed by the changes in the host, not by mutagenesis in virus. Inhibition of HCV RNA replication was observed by subjecting HCV replicon containing cell line to G418 and different concentrations of compound [32]. High SVR rate of nitazoxanide along with interferon suggested that nitazoxanide can be exercised instead of ribavirin to avoid side effect of this drug.

Another newly discovered antiviral compound, clemizole, is found to exhibit influential antiviral activity against NS4B RNA binding and HCV replication by using luciferase reporter-linked HCV replication assay. Clemizole has succumbed high synergistic effects with various protease (VX950 & SCH503034) and additive effects with polymerase inhibitors (NM283 & HCV796). Furthermore, the clemizole-SCH503034 combination reduces the manifestation of resistance exclusive of bestowing cross-resistance [33].

Cyclosporine A (CsA), an immunosuppressant for transplanted patients, has currently come forward as a forthcoming antiviral compound against HCV. It is evaluated that CsA persuasively inhibits HCV replication by illustrating the various HCV derived replicons with variable levels of CsA resistance due to mutations in NS5B. Transformed HCV replicons integrated with these mutations proved the resistance to CsA. Increased ability of mutant NS5B is associated with the enhanced binding to RNA in the presence of CsA and intramolecular interactions between the residues of thumb and C-terminal domains are crucial for HCV replicase function [34].

An innovative compound, ACH-806 (GS 9132) is characterized as antiviral agent against HCV by using HCV replicon system. ACH-806 was discovered by using HCV replicon cells [35]. Mechanism of action studies have exposed that ACH-806 averts the apposite pattern of replication complexes by sharply binding to NS4A [35]. Moreover, ACH-806 has been inveterated to decelerate HCV replication in genotype 1 HCV infected patients in clinical trial, while the reversible nephrotoxicity prohibits its additional clinical progress [36].

25-hydroxychloesterol (25-HC) has been ascertained as anti-HCV agent by modifying the mevalonate pathway [37]. Transcriptional profiling of 25-HC was executed on Huh-7 cells containing HCV replicons. Various sets of genes were up- and down regulated involved in the mevalonate pathway and instituted transcriptional changes resulting in the inhibition of HCV replication. The identified genes which may act as HCV markers are indirectly involved in the inhibition of HCV replication [38].

A class of anionic tetraphenylporphyrins is identified as explicit inhibitors of HCV replicons. *Meso*-tetrakis-(3, 5-dicarboxy-4,4'-biphenyl) porphyrin is found to display *in-vitro *antiviral activity against HCV genotype 1b replicons by targeting viral replicase but less proficient against the genotype 2a (JFH-1) replicon. Synergistic studies have shown that the combination of *Meso*-tetrakis-(3, 5-dicarboxy-4,4'-biphenyl) porphyrin with BILN 2061 and with IFN-α was additive to synergistic which lead to almost 90% inhibition of HCV replication [39].

TMC435350 is found to be a novel and specific protease inhibitor by establishing preclinical models and *in vitro *assays. TMC435350 is a potent HCV NS3/4A serine protease inhibitor which displays synergistic effects in combination with IFN-α and additive effects with RBV. Additionally, NS5B inhibitors NM-107 and HCV-796 in combination with TMC435350 showed synergism which debates the effectiveness of TMC435350 clinical antiviral therapy against HCV [40].

SCY-635 is a potent non-immunosuppressive disubstituted analogue of CsA showing evidence of antiviral activity against HCV by operating at host CyP, which is imperative for HCV RNA replication. SCY-635 stalled the peptidyl prolyl isomerase activity of CyP at nanomolar concentrations by testing in HCV replicon cell line. Further clinical trials of SCY-635 may prove to be beneficial in drug development for HCV in future [41]. Safety and pharmacokinetics of SCY-635 have also been studied in chronically HCV infected patients [42].

By doing *in-vitro *resistance study of AG-021541, it is being demonstrated that AG-021541 is a novel dihydropyrone NNI of HCV replication. AG-021541 marks to hit HCV RNA polymerase at the thumb-base allosteric site. As resistance changes due to AG-021541 remained entirely susceptible to IFN and polymerase inhibitors targeting sections distinct from the AG-021541 binding site. Due to lack of cross resistance, combinational therapy of AG-021541 with other polymerase or nonpolymerase inhibitors would be significantly accommodating in future [43].

ITMN-191 (R7227) is a peptidomimetic inhibitor of NS3/4A protease of HCV. ITMN-191 introverted a reference genotype 1 NS3/4A protein in a time-dependent manner, which is a characteristic of an inhibitor with a two-step binding mechanism and a low dissociation rate. Under pre-equilibrium circumstances, small quantity of ITMN-191 half-maximally inhibited the reference NS3/4A protease, but a 35,000-fold-higher concentration did not substantially restrain a group of 79 proteases, ion channels and transporters. Combinational therapeutic regime of ITMN-191 (R7227) is considered to be helpful in curing chronic hepatitis C [44].

GS-327073, 5-[{3-(4-chlorophenyl)-5-isoxazolyl} methyl]-2-(2, 3-difluorophenyl)-5H-imidazo [4,5-c] pyridine is proved to be highly effective against HCV replication by assessing in various HCV subgenomic replicons (genotypes 1b, 1a and 2a), in JFH-1 infectious system and against replicons which are sustained to be resistant for various HCV inhibitors. GS-327073, revealing pharmacokinetic characteristics *in-vitro *has maintained anti-HCV activity for resistant replicons [45].

P3 aza-peptide analogue (exhibiting anti HCV activity) of a novel HCV protease blocker (BILN 2061) has been synthesized. Anti HCV activity of newly synthesized derivative is shown to be less effective than the parent compound in HCV sub-genomic replicon assay. Configuration at P3 has interrupted the H-bond conformation which is necessary for the binding of compound to active site of HCV NS3 protease [46]. A series of gem-dialkyl naphthalenones have shown to exhibit antiviral activity against HCV. The extent of efficient inhibition activity is correlated with the length of carbon chain. Gem-dialkyl naphthalenone derivatives are found to be novel HCV polymerase inhibitors. By performing the modifications at carbon-1 of B ring, thriving results against HCV polymerase were attained in HCV sub-genomic replicons [47].

Novel sulfonamide P4-capped ketoamide second generation inhibitors of hepatitis C virus NS3 serine protease have been discovered. Discovery of one of them, showing potent anti HCV activity, is contributed by introducing the sulphonamide moiety and optimization of P1 residue. This potent inhibitor of HCV subgenomic replication reveals improved cellular potencies and good oral exposure in rat, dogs and monkey [48].

Telaprevir in combination with standard antiviral therapy against HCV bestowed rapid viral response and considerably declined the HCV RNA levels. Further, extensive studies are conducted to assess sustained virological response while administration of combinational therapy [49]. Telaprevir is the first drug against HCV presently in progress which exclusively blocks HCV NS3/4A serine protease.

A new series of geldanamycin (GA) derivatives have been synthesized which were evaluated as antiviral compounds against HCV in GS4.3 HCV replicon cells. Many of these synthesized compounds exhibited competitive anti-HCV activity [50].

**Figure 2:**
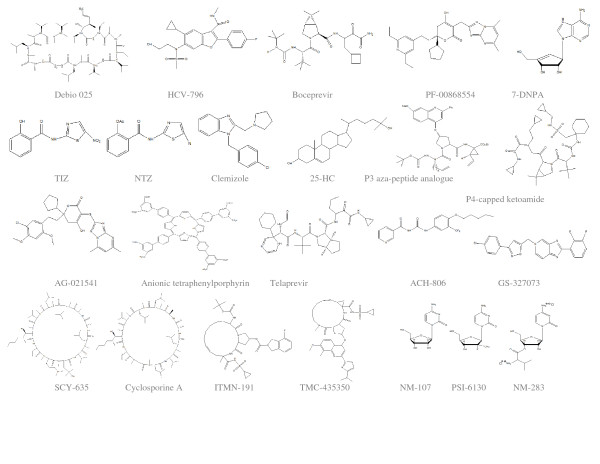
**Inhibitors of HCV replication**.

Various other novel HCV NS5B polymerase inhibitors have recently been discovered such as pyrano [3,4-b] indole based inhibitors, tricyclic 5,6-dihydro-1H-pyridin-2-ones, benzothiadiazine and 1,4-benzothiazine, 4-(1^/^,1^/^dioxo-1^/^dihydro-1^/^λ^6^-benzo [1^/^,2^/^,4^/^] thiadiazin-3^/^-yl)-5-hydroxy-2H-pyridazin-3-ones, Pyrrolo [1,2-b] pyridazin-2-ones, 2-(1,1-dioxo-2H-[1,2,4] benzothiadiazin-3-yl)-1-hydroxynaphthalene derivatives, pyrano [3,4-b] indole. (Structures are cited in figure 2).

### Helicase Inhibitors

NS3 helicase plays an important role in unwinding of double-strand DNA and duplex RNA. DEAD box proteins belong to helicase superfamily 2 that facilitate mRNA splicing, mRNA export, translation, protein processing, RNA packaging into virions, mitochondrial gene expression and probably aid RNA-dependent RNA replication [51-54]. DEAD-box stands for exceedingly conserved motif comprised of Asp-Glu-Ala-Asp. The two most striking targets on NS3 helicase are ATP and RNA binding sites while other distinctive facets may be utilized as target for drug development [55]. From a biological point of view, activities of protease and helicase co-exist *in-vivo*, thus may prove to be a useful antiviral target against HCV. Helicase and polymerase form viral helicase multi-protein complex. So, it is essential to inhibit functions that are fundamental for helicase activity.

Helicase inhibitors may act in different mechanisms such as by inhibiting NTPase activity, RNA binding and NTP hydrolysis coupling at the unwinding reaction.

A new series of compounds, acridone derivatives, were tested to measure inhibitory effects of derivatives against NS3 helicase activity of HCV in sub-genomic replicon assay. These substituted compounds were also investigated for transcription inhibition *in-vitro *based on the DNA-dependent T7 RNA polymerase. The majority of compounds were displayed as transcription inhibitors. Two compounds, *N*-(pyridin-4-yl)-amide and *N*-(pyridin-2-yl)-amide of acridone-4-carboxylic acid are competent RNA replication inhibitors verifying that the acridone derivatives may be deemed as impending antiviral mediator [56].

By employing helicase assays, 1-*N*, 4-*N*-bis [4-(1*H*-benzimidazol-2-yl) phenyl] benzene-1, 4-dicarboxamide ((BIP)_2 _B) is established to inhibit capability of HCV helicase to split double stranded DNA and RNA. (BIP)_2_B inhibited helicase-catalyzed ATP hydrolysis in the presence of RNA transitional concentrations, signifying RNA and (BIP)_2_B contend for alike binding site [55]. Helicase assay was performed to screen inhibitors by utilizing DOCK program. Fragment-based explorations were exploited to recognize triphenylmethane derivatives for other persuasive inhibitors. 3-bromo-4-hydroxyl substituted derivative masked HCV replication in the HCV replicon cells. For that reason, this inhibitor with structural novelty may act as a functional gibbet for the sighting of innovative HCV NS3 helicase inhibitors [57].

The most persuasive benzotriazole helicase inhibitors were recognized throughout the duration of random screening study [58,59]. In particular, 4, 5, 6, 7- tetrabromobenzotriazole (TBBT) acknowledged as a powerful and exceedingly discriminating inhibitor of protein kinase 2, which displayed inhibitory concentration (IC_50_) values of 20 *μ*M and 5,6-dichloro-1-(*β*-D-ribofuranosyl) benzotriazole (DRBT) demonstrated IC_50 _values of 1.5 *μ*M.

The most active chemical entity, 3, 5, 7-tri [(40-methylpiperazin-10-yl) methyl] tropolone inhibited RNA replication by 50% at an effective concentration (EC_50_) of 46.9 μM, while the most competent one was 3, 5, 7-tri [(30-methylpiperidin-10-yl) methyl] tropolone having EC_50 _of 35.6 μM. These derivatives are the first helicase inhibitors that block replication of HCV with the capability of causing the emergence of resistant mutants [60].

Another HCV helicase inhibitor, QU663, illustrated discriminating inhibition without disturbing NS3 helicase hydrolysis potential. QU663 might function as a potent inhibitor with respect to nucleic acid substrate by lessening the likeness of the enzyme for the substrate. QU663 blocks NS3 unwinding activity, thus making it a potential competitor for antiviral drugs against HCV [61].

**Figure 3:**
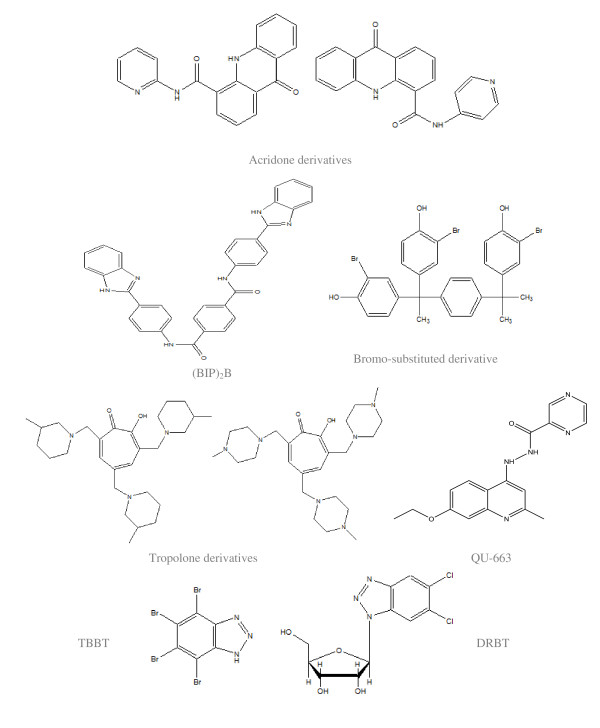
**Inhibitors of HCV helicase**.

Two series of compounds exhibiting aminophenylbenzimidazole and benzimidazole like entities are patented by ViroPharma Inc. as HCV helicase inhibitors [62]. Vertex Pharmaceuticals Inc. accounted various aminothiadiazoliums which also exhibit anti-helicase activity but with lower efficacy [63]. Two derivatives of 2-arylbenzofuran isolated from Mori cortex radicis have shown potent inhibition against HCV NS3 helicase [62]. (Structures are cited in figure 3).

### Inhibitors of HCV Entry

For the development of antiviral drugs against HCV entry, enveloped proteins have been extensively utilized, especially targeting the carbohydrate moieties on E1 and E2 proteins. The first step of HCV life cycle involves the attachment of viral particles to the cell surface which is followed by internalization. So, various entry inhibitors are reported to prevent the entry of virions.

PD 404, 182, primarily a bacterial KDO 8-P synthase inhibitor, has revealed the restraining of HCV pseudoparticles (HCVpp) and VSV-Gpp entry in a dose-dependent manner, which signifies the hindrance with a process entailed for the HCVpp entry [71]. Fluphenazine, PCperazine, and trifluoperazine were currently recognized as inhibitors of HCV entry [64]. These compounds alienated the D2 and D1 dopamine [65,66] and 5-HT2 serotonin receptors [67] in neural signaling networks.

**Figure 4:**
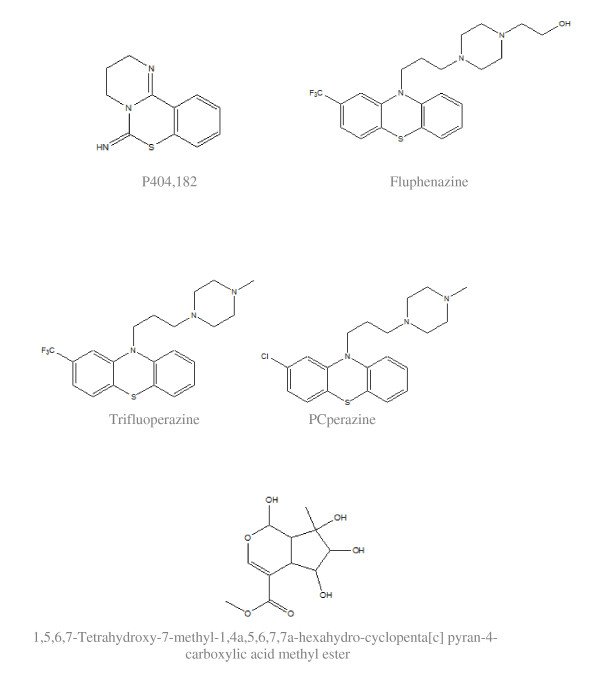
**Inhibitors of HCV entry**.

A series of iridoids from Lamium album have been appraised for their efficiency in blocking HCV cell entry and HCVpp infection. The occurrence of the anti-HCV iridoid aglycone epimers, lamiridosins A/B (1/2), in the primed aqueous extract of Lamium album, have shown the diminution in HCVpp entry due to interruption in the binding of HCV E2 with CD81 receptor [68]. (Structures are cited in figure 4).

## Conclusion

More importantly, it is crucial to appraise *in-vitro *combinational therapy of small inhibitory molecules with standard regime to improve antiviral activity against HCV replication and infection. Therapeutic drugs against HCV may have the potential to put off the replication complex formation [37], to inhibit host cell kinases [69], to block protein folding pathways [70] and targeting to hormone receptors [71]. Accordingly, therapeutic regime for HCV have been insinuating in a novel trail with less side effects and more efficacy than standard therapy. Consequently, compounds that may change any mechanism of cell regulation which is provoked by HCV can have the propensity to alleviate the infection. Various inhibitors are now crossing the threshold in human clinical trials in different phases such as BILN 2061, ITMN 191, TMC 435350, MK 7009 (I & II phase) and α-ketoamide (phase III) etc. For drug designing, main emphasis is made on three major targets but NS3 protease inhibitors are the most successful one. But unfortunately various drugs exhibit propensity to resistance emergence. In order to avoid such problem, there is a need to develop other potential antiviral drugs. So, natural products should be included especially in combinational therapy which may prove to be a better treatment option than standard therapy.

## Competing interests

The authors declare that they have no competing interests.

## Authors' contributions

SDR and UAA contributed equally in manuscript design and write up. All the authors read and approved the final manuscript.

## References

[B1] Baldo V Baldovin T Trivello R Floreani A Epidemiology of HCV infection Curr Pharm Des 2008 14 1646 1654 10.2174/138161208784746770 18673187

[B2] Raja NS Janjua NK Epidemiology of hepatitis C virus infection in Pakistan. J Microbiol Immunol Infect J Microbiol Immunol Infect 2008 41 4 8 18327420

[B3] Choo QL Kuo G Weiner AJ Overby LR Bradley DW Houghton M Isolation of a cDNA clone derived from a blood-borne non-A, non-B viral hepatitis genome Science 1989 244 359 362 10.1126/science.2523562 2523562

[B4] Reed KE Rice CM Overview of hepatitis C virus genome structure, polyprotein processing, and protein properties Curr Top Microbiol Immunol 2000 242 55 84 10592656 10.1007/978-3-642-59605-6_4

[B5] Pinzani M Vizzutti F Arena U Marra F Technology Insight: noninvasive assessment of liver fibrosis by biochemical scores and elastography Nat Clin Pract Gastroenterol Hepatol 2008 5 95 106 10.1038/ncpgasthep1025 18253138

[B6] Friedman SL Liver fibrosis -- from bench to bedside J Hepatol 2003 38 Suppl 1 S38 53 12591185 10.1016/s0168-8278(02)00429-4

[B7] Poynard T Ratziu V McHutchison J Manns M Goodman Z Zeuzem S Younossi Z Albrecht J Effect of treatment with peginterferon or interferon alfa-2b and ribavirin on steatosis in patients infected with hepatitis C Hepatology 2003 38 75 85 12829989 10.1053/jhep.2003.50267

[B8] Burlone ME Budkowska A Hepatitis C virus cell entry: role of lipoproteins and cellular receptors J Gen Virol 2009 90 1055 1070 10.1099/vir.0.008300-0 19264629

[B9] Blanchard E Belouzard S Goueslain L Wakita T Dubuisson J Wychowski C Rouille Y Hepatitis C virus entry depends on clathrin-mediated endocytosis J Virol 2006 80 6964 6972 10.1128/JVI.00024-06 16809302 PMC1489042

[B10] Meertens L Bertaux C Dragic T Hepatitis C virus entry requires a critical postinternalization step and delivery to early endosomes via clathrin-coated vesicles J Virol 2006 80 11571 11578 10.1128/JVI.01717-06 17005647 PMC1642584

[B11] Feld JJ Hoofnagle JH Mechanism of action of interferon and ribavirin in treatment of hepatitis C Nature 2005 436 967 972 10.1038/nature04082 16107837

[B12] Zeuzem S Feinman SV Rasenack J Heathcote EJ Lai MY Gane E O'Grady J Reichen J Diago M Lin A Peginterferon alfa-2a in patients with chronic hepatitis C N Engl J Med 2000 343 1666 1672 10.1056/NEJM200012073432301 11106715

[B13] De Francesco R Migliaccio G Challenges and successes in developing new therapies for hepatitis C Nature 2005 436 953 960 10.1038/nature04080 16107835

[B14] Hao W Herlihy KJ Zhang NJ Fuhrman SA Doan C Patick AK Duggal R Development of a novel dicistronic reporter-selectable hepatitis C virus replicon suitable for high-throughput inhibitor screening Antimicrob Agents Chemother 2007 51 95 102 10.1128/AAC.01008-06 17060518 PMC1797688

[B15] Tedesco R Shaw AN Bambal R Chai D Concha NO Darcy MG Dhanak D Fitch DM Gates A Gerhardt WG 3-(1,1-dioxo-2H-(1,2,4)-benzothiadiazin-3-yl)-4-hydroxy-2(1H)-quinolinones, potent inhibitors of hepatitis C virus RNA-dependent RNA polymerase J Med Chem 2006 49 971 983 10.1021/jm050855s 16451063

[B16] Zuck P Murray EM Stec E Grobler JA Simon AJ Strulovici B Inglese J Flores OA Ferrer M A cell-based beta-lactamase reporter gene assay for the identification of inhibitors of hepatitis C virus replication Anal Biochem 2004 334 344 355 10.1016/j.ab.2004.07.031 15494142

[B17] Wang C Gale M Jr Keller BC Huang H Brown MS Goldstein JL Ye J Identification of FBL2 as a geranylgeranylated cellular protein required for hepatitis C virus RNA replication Mol Cell 2005 18 425 434 10.1016/j.molcel.2005.04.004 15893726

[B18] Watashi K Ishii N Hijikata M Inoue D Murata T Miyanari Y Shimotohno K Cyclophilin B is a functional regulator of hepatitis C virus RNA polymerase Mol Cell 2005 19 111 122 10.1016/j.molcel.2005.05.014 15989969

[B19] Zhang J Yamada O Sakamoto T Yoshida H Iwai T Matsushita Y Shimamura H Araki H Shimotohno K Down-regulation of viral replication by adenoviral-mediated expression of siRNA against cellular cofactors for hepatitis C virus Virology 2004 320 135 143 10.1016/j.virol.2003.11.023 15003869

[B20] Al RH Xie Y Wang Y Hagedorn CH Expression of recombinant hepatitis C virus non-structural protein 5B in Escherichia coli Virus Res 1998 53 141 149 10.1016/S0168-1702(97)00147-0 9620206

[B21] Ferrari E Wright-Minogue J Fang JW Baroudy BM Lau JY Hong Z Characterization of soluble hepatitis C virus RNA-dependent RNA polymerase expressed in Escherichia coli J Virol 1999 73 1649 1654 9882374 10.1128/jvi.73.2.1649-1654.1999 PMC103993

[B22] Sampath A Padmanabhan R Molecular targets for flavivirus drug discovery Antiviral Res 2009 81 6 15 10.1016/j.antiviral.2008.08.004 18796313 PMC2647018

[B23] Bartenschlager R The hepatitis C virus replicon system: from basic research to clinical application J Hepatol 2005 43 210 216 10.1016/j.jhep.2005.05.013 15964655

[B24] Coelmont L Kaptein S Paeshuyse J Vliegen I Dumont JM Vuagniaux G Neyts J Debio 025, a cyclophilin binding molecule, is highly efficient in clearing hepatitis C virus (HCV) replicon-containing cells when used alone or in combination with specifically targeted antiviral therapy for HCV (STAT-C) inhibitors Antimicrob Agents Chemother 2009 53 967 976 10.1128/AAC.00939-08 19104013 PMC2650540

[B25] Coelmont L Hanoulle X Chatterji U Berger C Snoeck J Bobardt M Lim P Vliegen I Paeshuyse J Vuagniaux G DEB025 (Alisporivir) inhibits hepatitis C virus replication by preventing a cyclophilin A induced cis-trans isomerisation in domain II of NS5A PLoS One 5 e13687 10.1371/journal.pone.0013687 PMC2965138 21060866

[B26] Flint M Mullen S Deatly AM Chen W Miller LZ Ralston R Broom C Emini EA Howe AY Selection and characterization of hepatitis C virus replicons dually resistant to the polymerase and protease inhibitors HCV-796 and boceprevir (SCH 503034) Antimicrob Agents Chemother 2009 53 401 411 10.1128/AAC.01081-08 18936191 PMC2630598

[B27] Ludmerer SW Graham DJ Boots E Murray EM Simcoe A Markel EJ Grobler JA Flores OA Olsen DB Hazuda DJ LaFemina RL Replication fitness and NS5B drug sensitivity of diverse hepatitis C virus isolates characterized by using a transient replication assay Antimicrob Agents Chemother 2005 49 2059 2069 10.1128/AAC.49.5.2059-2069.2005 15855532 PMC1087645

[B28] Tripathi RL Krishnan P He Y Middleton T Pilot-Matias T Chen CM Lau DT Lemon SM Mo H Kati W Molla A Replication efficiency of chimeric replicon containing NS5A-5B genes derived from HCV-infected patient sera Antiviral Res 2007 73 40 49 10.1016/j.antiviral.2006.07.005 16914212

[B29] Shi ST Herlihy KJ Graham JP Nonomiya J Rahavendran SV Skor H Irvine R Binford S Tatlock J Li H Preclinical characterization of PF-00868554, a potent nonnucleoside inhibitor of the hepatitis C virus RNA-dependent RNA polymerase Antimicrob Agents Chemother 2009 53 2544 2552 10.1128/AAC.01599-08 19307358 PMC2687230

[B30] Kim HJ Sharon A Bal C Wang J Allu M Huang Z Murray MG Bassit L Schinazi RF Korba B Chu CK Synthesis and anti-hepatitis B virus and anti-hepatitis C virus activities of 7-deazaneplanocin A analogues in vitro J Med Chem 2009 52 206 213 10.1021/jm801418v 19072694 PMC2725430

[B31] Bassit L Grier J Bennett M Schinazi RF Combinations of 2'-C-methylcytidine analogues with interferon-alpha2b and triple combination with ribavirin in the hepatitis C virus replicon system Antivir Chem Chemother 2008 19 25 31 18610555 10.1177/095632020801900104 PMC2742417

[B32] Korba BE Elazar M Lui P Rossignol JF Glenn JS Potential for hepatitis C virus resistance to nitazoxanide or tizoxanide Antimicrob Agents Chemother 2008 52 4069 4071 10.1128/AAC.00078-08 18710916 PMC2573111

[B33] Einav S Sobol HD Gehrig E Glenn JS The hepatitis C virus (HCV) NS4B RNA binding inhibitor clemizole is highly synergistic with HCV protease inhibitors J Infect Dis 202 65 74 10.1086/653080 PMC3008401 20486856

[B34] Liu Z Robida JM Chinnaswamy S Yi G Robotham JM Nelson HB Irsigler A Kao CC Tang H Mutations in the hepatitis C virus polymerase that increase RNA binding can confer resistance to cyclosporine A Hepatology 2009 50 25 33 10.1002/hep.22987 19489073 PMC2727352

[B35] Yang W Zhao Y Fabrycki J Hou X Nie X Sanchez A Phadke A Deshpande M Agarwal A Huang M Selection of replicon variants resistant to ACH-806, a novel hepatitis C virus inhibitor with no cross-resistance to NS3 protease and NS5B polymerase inhibitors Antimicrob Agents Chemother 2008 52 2043 2052 10.1128/AAC.01548-07 18411324 PMC2415816

[B36] Pottage JC Lawitz E Mazur D Wyles H Vargas R Ghalib R Gugliotti M Donohue aHR Short-term antiviral activity and safety of ACH-806 (GS-9132), an NS4A antagonist, in HCV genotype 1 infected individuals J Hepatol 2007 46 Suppl 1 A783

[B37] Sagan SM Rouleau Y Leggiadro C Supekova L Schultz PG Su AI Pezacki JP The influence of cholesterol and lipid metabolism on host cell structure and hepatitis C virus replication Biochem Cell Biol 2006 84 67 79 10.1139/o05-149 16462891

[B38] Pezacki JP Sagan SM Tonary AM Rouleau Y Belanger S Supekova L Su AI Transcriptional profiling of the effects of 25-hydroxycholesterol on human hepatocyte metabolism and the antiviral state it conveys against the hepatitis C virus BMC Chem Biol 2009 9 2 10.1186/1472-6769-9-2 19149867 PMC2651120

[B39] Cheng Y Tsou LK Cai J Aya T Dutschman GE Gullen EA Grill SP Chen AP Lindenbach BD Hamilton AD Cheng YC A novel class of meso-tetrakis-porphyrin derivatives exhibits potent activities against hepatitis C virus genotype 1b replicons in vitro Antimicrob Agents Chemother 54 197 206 10.1128/AAC.01206-09 PMC2798527 19901090

[B40] Lin TI Lenz O Fanning G Verbinnen T Delouvroy F Scholliers A Vermeiren K Rosenquist A Edlund M Samuelsson B In vitro activity and preclinical profile of TMC435350, a potent hepatitis C virus protease inhibitor Antimicrob Agents Chemother 2009 53 1377 1385 10.1128/AAC.01058-08 19171797 PMC2663092

[B41] Hopkins S Scorneaux B Huang Z Murray MG Wring S Smitley C Harris R Erdmann F Fischer G Ribeill Y SCY-635, a novel nonimmunosuppressive analog of cyclosporine that exhibits potent inhibition of hepatitis C virus RNA replication in vitro Antimicrob Agents Chemother 54 660 672 10.1128/AAC.00660-09 PMC2812147 19933795

[B42] Hopkins S Heuman D Gavis E Lalezari J Glutzer E DiMassimo B Rusnak P Wring S Safety, plasma, pharmacokinetics, and anti-viral activity of SCY-635 in adult patients with chronic hepatitis C virus infection J Hepatol 2009 50 Suppl 1 S36 Smitley SaRYS, plasma, pharmacokinetics, and anti-viral activity of SCY-635 in adult patients with chronic hepatitis C virus infection

[B43] Shi ST Herlihy KJ Graham JP Fuhrman SA Doan C Parge H Hickey M Gao J Yu X Chau F In vitro resistance study of AG-021541, a novel nonnucleoside inhibitor of the hepatitis C virus RNA-dependent RNA polymerase Antimicrob Agents Chemother 2008 52 675 683 10.1128/AAC.00834-07 18070954 PMC2224781

[B44] Seiwert SD Andrews SW Jiang Y Serebryany V Tan H Kossen K Rajagopalan PT Misialek S Stevens SK Stoycheva A Preclinical characteristics of the hepatitis C virus NS3/4A protease inhibitor ITMN-191 (R7227) Antimicrob Agents Chemother 2008 52 4432 4441 10.1128/AAC.00699-08 18824605 PMC2592891

[B45] Vliegen I Paeshuyse J De Burghgraeve T Lehman LS Paulson M Shih IH Mabery E Boddeker N De Clercq E Reiser H Substituted imidazopyridines as potent inhibitors of HCV replication J Hepatol 2009 50 999 1009 10.1016/j.jhep.2008.12.028 19303654 PMC7114863

[B46] Randolph JT Zhang X Huang PP Klein LL Kurtz KA Konstantinidis AK He W Kati WM Kempf DJ Synthesis, antiviral activity, and conformational studies of a P3 aza-peptide analog of a potent macrocyclic tripeptide HCV protease inhibitor Bioorg Med Chem Lett 2008 18 2745 2750 10.1016/j.bmcl.2008.02.053 18375121

[B47] Bosse TD Larson DP Wagner R Hutchinson DK Rockway TW Kati WM Liu Y Masse S Middleton T Mo H Synthesis and SAR of novel 1,1-dialkyl-2(1H)-naphthalenones as potent HCV polymerase inhibitors Bioorg Med Chem Lett 2008 18 568 570 10.1016/j.bmcl.2007.11.088 18068361

[B48] Bogen SL Arasappan A Velazquez F Blackman M Huelgas R Pan W Siegel E Nair LG Venkatraman S Guo Z Discovery of potent sulfonamide P4-capped ketoamide second generation inhibitors of hepatitis C virus NS3 serine protease with favorable pharmacokinetic profiles in preclinical species Bioorg Med Chem 18 1854 1865 10.1016/j.bmc.2010.01.044 20149666

[B49] Lawitz E Rodriguez-Torres M Muir AJ Kieffer TL McNair L Khunvichai A McHutchison JG Antiviral effects and safety of telaprevir, peginterferon alfa-2a, and ribavirin for 28 days in hepatitis C patients J Hepatol 2008 49 163 169 10.1016/j.jhep.2008.03.027 18486984

[B50] Shan GZ Peng ZG Li YH Li D Li YP Meng S Gao LY Jiang JD Li ZR A novel class of geldanamycin derivatives as HCV replication inhibitors targeting on Hsp90: synthesis, structure-activity relationships and anti-HCV activity in GS4.3 replicon cells J Antibiot (Tokyo) 64 177 182 10.1038/ja.2010.161 21179047

[B51] Lorsch JR RNA chaperones exist and DEAD box proteins get a life Cell 2002 109 797 800 10.1016/S0092-8674(02)00804-8 12110176

[B52] Tanner NK Linder P DExD/H box RNA helicases: from generic motors to specific dissociation functions Mol Cell 2001 8 251 262 10.1016/S1097-2765(01)00329-X 11545728

[B53] Linder P Stutz F mRNA export: travelling with DEAD box proteins Curr Biol 2001 11 R961 963 10.1016/S0960-9822(01)00574-7 11728322

[B54] de la Cruz J Kressler D Linder P Unwinding RNA in Saccharomyces cerevisiae: DEAD-box proteins and related families Trends Biochem Sci 1999 24 192 198 10.1016/S0968-0004(99)01376-6 10322435

[B55] Belon CA High YD Lin TI Pauwels F Frick DN Mechanism and specificity of a symmetrical benzimidazolephenylcarboxamide helicase inhibitor Biochemistry 49 1822 1832 10.1021/bi901974a PMC2832472 20108979

[B56] Stankiewicz-Drogon A Palchykovska LG Kostina VG Alexeeva IV Shved AD Boguszewska-Chachulska AM New acridone-4-carboxylic acid derivatives as potential inhibitors of hepatitis C virus infection Bioorg Med Chem 2008 16 8846 8852 10.1016/j.bmc.2008.08.074 18801660

[B57] Chen CS Chiou CT Chen GS Chen SC Hu CY Chi WK Chu YD Hwang LH Chen PJ Chen DS Structure-based discovery of triphenylmethane derivatives as inhibitors of hepatitis C virus helicase J Med Chem 2009 52 2716 2723 10.1021/jm8011905 19419203

[B58] Borowski P Deinert J Schalinski S Bretner M Ginalski K Kulikowski T Shugar D Halogenated benzimidazoles and benzotriazoles as inhibitors of the NTPase/helicase activities of hepatitis C and related viruses Eur J Biochem 2003 270 1645 1653 10.1046/j.1432-1033.2003.03540.x 12694177

[B59] Bretner M Baier A Kopanska K Najda A Schoof A Reinholz M Lipniacki A Piasek A Kulikowski T Borowski P Synthesis and biological activity of 1H-benzotriazole and 1H-benzimidazole analogues--inhibitors of the NTpase/helicase of HCV and of some related Flaviviridae Antivir Chem Chemother 2005 16 315 326 16245647 10.1177/095632020501600504

[B60] Najda-Bernatowicz A Krawczyk M Stankiewicz-Drogon A Bretner M Boguszewska-Chachulska AM Studies on the anti-hepatitis C virus activity of newly synthesized tropolone derivatives: identification of NS3 helicase inhibitors that specifically inhibit subgenomic HCV replication Bioorg Med Chem 18 5129 5136 10.1016/j.bmc.2010.05.066 20579888

[B61] Maga G Gemma S Fattorusso C Locatelli GA Butini S Persico M Kukreja G Romano MP Chiasserini L Savini L Specific targeting of hepatitis C virus NS3 RNA helicase. Discovery of the potent and selective competitive nucleotide-mimicking inhibitor QU663 Biochemistry 2005 44 9637 9644 10.1021/bi047437u 16008349

[B62] Lee HY Yum JH Rho YK Oh SJ Choi HS Chang HB Choi DH Leem MJ Choi EJ Ryu JM Hwang SB Inhibition of HCV replicon cell growth by 2-arylbenzofuran derivatives isolated from Mori Cortex Radicis Planta Med 2007 73 1481 1485 10.1055/s-2007-990249 17948170

[B63] Chockalingam K Simeon RL Rice CM Chen Z A cell protection screen reveals potent inhibitors of multiple stages of the hepatitis C virus life cycle Proc Natl Acad Sci USA 107 3764 3769 10.1073/pnas.0915117107 PMC2840489 20142494

[B64] Gastaminza P Whitten-Bauer C Chisari FV Unbiased probing of the entire hepatitis C virus life cycle identifies clinical compounds that target multiple aspects of the infection Proc Natl Acad Sci USA 107 291 296 10.1073/pnas.0912966107 PMC2806752 19995961

[B65] Cai G Gurdal H Smith C Wang HY Friedman E Inverse agonist properties of dopaminergic antagonists at the D(1A) dopamine receptor: uncoupling of the D(1A) dopamine receptor from G(s) protein Mol Pharmacol 1999 56 989 996 10531405 10.1124/mol.56.5.989

[B66] Lummis SC Baker J Radioligand binding and photoaffinity labelling studies show a direct interaction of phenothiazines at 5-HT3 receptors Neuropharmacology 1997 36 665 670 10.1016/S0028-3908(97)00054-3 9225292

[B67] Herrick-Davis K Grinde E Teitler M Inverse agonist activity of atypical antipsychotic drugs at human 5-hydroxytryptamine2C receptors J Pharmacol Exp Ther 2000 295 226 232 10991983

[B68] Zhang H Rothwangl K Mesecar AD Sabahi A Rong L Fong HH Lamiridosins, hepatitis C virus entry inhibitors from Lamium album J Nat Prod 2009 72 2158 2162 10.1021/np900549e 19904996

[B69] Rakic B Clarke J Tremblay TL Taylor J Schreiber K Nelson KM Abrams SR Pezacki JP A small-molecule probe for hepatitis C virus replication that blocks protein folding Chem Biol 2006 13 1051 1060 10.1016/j.chembiol.2006.08.010 17052609

[B70] Supekova L Supek F Lee J Chen S Gray N Pezacki JP Schlapbach A Schultz PG Identification of human kinases involved in hepatitis C virus replication by small interference RNA library screening J Biol Chem 2008 283 29 36 17951261 10.1074/jbc.M703988200

[B71] Rakic B Sagan SM Noestheden M Belanger S Nan X Evans CL Xie XS Pezacki JP Peroxisome proliferator-activated receptor alpha antagonism inhibits hepatitis C virus replication Chem Biol 2006 13 23 30 10.1016/j.chembiol.2005.10.006 16426968

